# A Case of Palytoxin Poisoning in a Home Aquarium Enthusiast and His Family

**DOI:** 10.1155/2015/621815

**Published:** 2015-10-26

**Authors:** Christine Hall, David Levy, Steven Sattler

**Affiliations:** Emergency Medicine Residency Program, Good Samaritan Hospital Medical Center, West Islip, NY 11795, USA

## Abstract

Inhalational exposure to palytoxin is an extremely rare cause of respiratory distress. This little-known marine toxin has the potential to cause significant morbidity and mortality. Toxicity has been best documented in cases of ingestion but has also been seen in cases of dermal exposure and inhalation of vapors. Palytoxin has been found in several coral species, some of which are favored by home aquarium enthusiasts and are commercially available. We report a case of a family who were exposed to the aerosolized toxin following the cleaning of a coral in their home aquarium. It is important that clinicians be aware of this source of toxic exposure to provide necessary care to these patients.

## 1. Introduction

**Figure 1:**
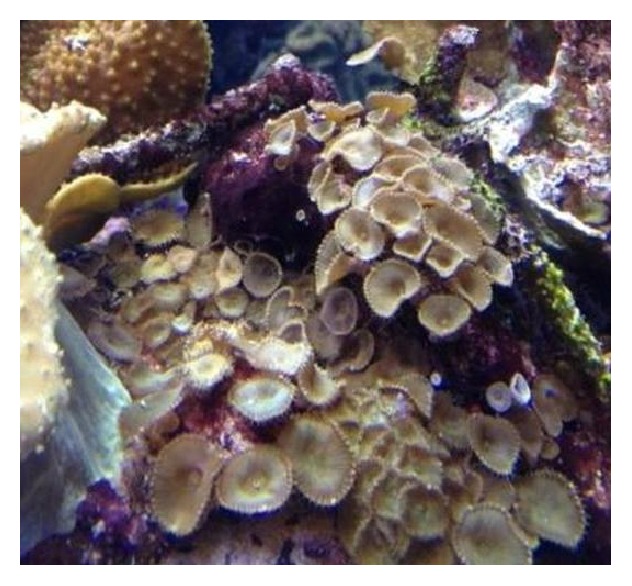
Coral similar to that encountered by the patient.

Palytoxin is a highly toxic substance and has been isolated from certain marine species including Zoantharia coral (Figure 1). This particular species is available to those who collect coral for home aquariums. In this case a 53-year-old male presented to the Emergency Department (ED) with dyspnea, starting shortly after cleaning his exotic coral species from his home aquarium which he identified as a Zoantharia species. A literature review identified only a limited number of cases of inhalational exposure attributable to palytoxin, although there is an abundance of self-reported exposures found on the Internet.

## 2. Case Report

A 53-year-old male presented to the ED with his wife and followed shortly by their daughter for evaluation of dyspnea which began approximately six hours prior to arrival. He had an associated nonproductive cough which had been worsening over that time period. He reported a subjective fever, chills, and myalgias. He had taken Robitussin and Aleve prior to arrival without relief. He stated that the symptoms began 1-2 hours following the cleaning of an exotic coral which he identified as a species of Zoantharia from his home aquarium. He had cleaned the coral using hot tap water in his basement sink. He identified the coral as a Zoantharia species of coral. He had not previously cleaned that particular coral and did not use any protective equipment while cleaning it. Both his wife and daughter were present in the home while he was cleaning the coral. His wife was in an adjoining basement room and his daughter was upstairs on the first floor of the home. Both presented to the ED with similar but less severe symptoms with the degree of severity commensurate with the distance from the location where the coral was being cleaned.

The patient's past medical history was significant only for hypothyroidism, hyperlipidemia, and psoriasis for which he was taking levothyroxine, rosuvastatin, and glucosamine chondroitin. Social history was significant for prior tobacco use, with a 20 pack-year history, with cessation one year prior to this encounter.

Vital signs included tachycardia at a rate of 112, a blood pressure of 155/83, respiratory rate of 18 with an oxygen saturation of 96% at room air, and a temperature of 102.6°F taken orally. On physical examination, bilateral expiratory wheezes with normal chest excursion were noted, with normal chest excursion. The patient had no evidence of respiratory distress. Cardiac examination revealed a regular tachycardia without murmurs, rubs, or gallops. The patient's wife's and daughter's examination revealed similar findings.

Acetaminophen was given for the fever with improvement of temperature to 100.8°F. Nebulized albuterol was given for the dyspnea and wheezing, with minimal improvement of symptoms. A portable chest X-ray was obtained and was normal. An EKG showed sinus tachycardia. Lab findings were significant for leukocytosis of 14,000, and an influenza swab was negative for influenza A and influenza B, and cardiac troponin I and brain natriuretic peptide (BNP) were normal.

**Figure 2:**
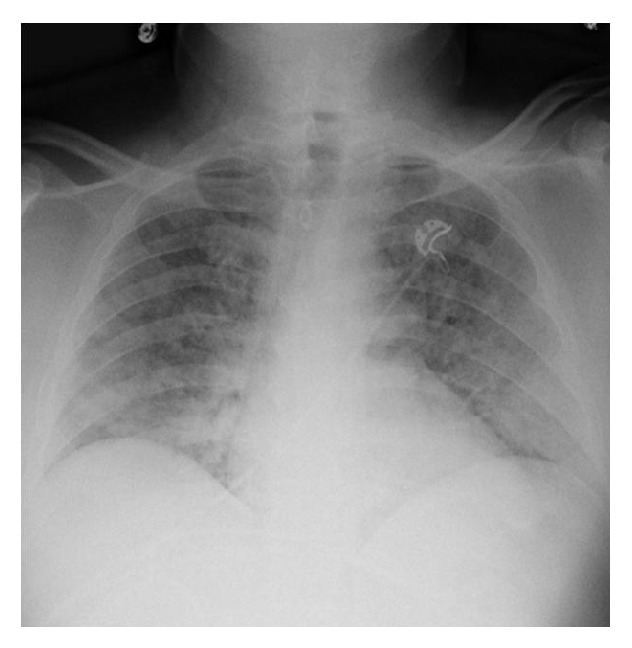
Chest X-ray from hospital day 3 showing worsening bibasilar opacities.

The patient's condition continued to deteriorate requiring increasing supplemental oxygen to maintain his saturation above 90%. He received repeated doses of nebulized albuterol and oral acetaminophen with no change in condition. An arterial blood gas (ABG) was obtained while the patient was receiving four liters of oxygen by nasal cannula that was significant for a partial oxygen pressure (pO_2_) of 65 mmHg. The New York City Poison Control Center was consulted and reported that certain toxic coral species have been found to cause pulmonary edema in cases of inhalational exposure when being handled out of water. As a result of the worsening clinical manifestations the patient was admitted to the intensive care unit with a diagnosis of respiratory distress from inhalational palytoxin exposure. During the course of the hospitalization the patient experienced a worsening cough, increased generalized weakness, and malaise. On the second inpatient day he developed hemoptysis. His pO_2_ on ABG decreased further to 76 mmHg while on 85% FiO_2_ via face mask. Serial chest X-rays demonstrated worsening bibasilar opacities (Figure 2). Lab work was significant for continued leukocytosis peaking at 22,000 on the third inpatient day. An echocardiogram was performed with no significant cardiac dysfunction identified. By day 4, the patient began to demonstrate mild clinical improvement with decreasing hemoptysis and improvement of generalized weakness. He continued to require supplemental oxygen by face mask throughout his hospitalization, with O_2_ saturations dropping to the mid-80s with trials at room air.

After seven days of inpatient treatment the patient was discharged home with the diagnosis of acute toxic lung injury. At the time of discharge he required portable oxygen. He was also discharged with an albuterol metered dose inhaler and prednisone taper. The patient's wife and daughter were also hospitalized at the initial ED encounter but clinically improved after 24 hours and were discharged home in stable condition after symptomatic treatment and observation. The patient continued to require portable O_2_ by nasal cannula and nebulized albuterol for 1 month following discharge, following which he made a complete recovery.

## 3. Discussion

Palytoxin is known to be a dangerous and often deadly toxin which can cause significant morbidity and mortality [1, 2]. Cases of human exposure have been documented from ingestion of contaminated seafood, dermal exposure, and inhalational exposure [3, 4]. This rare toxin which is most often found in soft corals and dinoflagellates has been definitively identified in zoanthid corals found both in the homes of collectors and for sale commercially [2].

Palytoxin is a marine polyether toxin which has effects on the cellular level. The toxin targets the sodium-potassium ATPase pumps inhibiting their function [4–6]. Human fatalities have been reported in cases of palytoxin ingestion [5]. In these cases mortality occurred primarily from myocardial damage and rhabdomyolysis resulting in renal failure [1].

One of the largest and earliest documented cases of inhalation exposures linked to palytoxin was reported during an* Ostreopsis ovata* bloom in the Mediterranean in 2006. It affected over 200 individuals in the local area. Exposure caused a spectrum of illness from mild symptoms such as rhinorrhea, cough, dyspnea, fever, and bronchospasm, to more severe illness in some of those exposed, requiring hospitalization and supportive care in approximately 10% of those reporting symptoms [2].

In recent literature, an increasing number of cases of home inhalational exposures have been reported [7–10]. The aerosolized toxin has affected entire families following exposure to Zoantharia coral in the home. To date, there have been no documented fatalities from inhalational exposure to palytoxin. These patients have frequently required hospitalization and supportive care for mild to severe respiratory reactions. The most commonly reported presenting symptoms are fever, cough, and dyspnea. Some patients additionally presented with chest pain and headache. Patients report a sudden onset of symptoms within minutes to hours after exposure to the coral species during cleaning or following attempted destruction of the coral with hot or boiling water. On presentation most patients were found to be febrile, tachycardic, and tachypneic, and in some cases wheezes were noted on physical examination. Lab findings of leukocytosis have been consistently reported in cases of inhalational exposure. Treatment in all of these cases was supportive, primarily with inhaled corticosteroids [7–10]. Most patients were discharged from the hospital following a short period of observation, although some patients with more severe respiratory symptoms required hospitalization [7].

Inhalational exposure to palytoxin is an extremely rare cause of respiratory distress. Although there is currently no definitive test to diagnose palytoxin exposure, a similar constellation of symptoms and laboratory findings have been described in previously reported inhalational exposures attributed to Zoantharia coral species. The presentation of this patient and his family and the synchronicity of symptomatic onset are highly indicative of a toxic inhalational exposure from the coral species in their home aquarium.
